# Light-Dependent Development of Circadian Gene Expression in Transgenic Zebrafish

**DOI:** 10.1371/journal.pbio.0030034

**Published:** 2005-02-01

**Authors:** Maki Kaneko, Gregory M Cahill

**Affiliations:** **1**Department of Biology and Biochemistry, University of HoustonTexasUnited States of America; University of GenevaSwitzerland

## Abstract

The roles of environmental stimuli in initiation and synchronization of circadian oscillation during development appear to vary among different rhythmic processes. In zebrafish, a variety of rhythms emerge in larvae only after exposure to light-dark (LD) cycles, whereas zebrafish *period3 (per3)* mRNA has been reported to be rhythmic from day 1 of development in constant conditions. We generated transgenic zebrafish in which expression of the firefly *luciferase (luc)* gene is driven by the zebrafish *per3* promoter. Live larvae from these lines are rhythmically bioluminescent, providing the first vertebrate system for high-throughput measurement of circadian gene expression in vivo. Circadian rhythmicity in constant conditions was observed only after 5–6 d of development, and only if the fish were exposed to LD signals after day 4. Regardless of light exposure, a novel developmental profile was observed, with low expression during the first few days and a rapid increase when active swimming begins. Ambient temperature affected the developmental profile and overall levels of *per3* and *luc* mRNA, as well as the critical days in which LD cycles were needed for robust bioluminescence rhythms. In summary, *per3-luc* zebrafish has revealed complex interactions among developmental events, light, and temperature in the expression of a clock gene.

## Abstract 2

Generation of zebrafish that express the firefly *luciferase* gene under a clock gene promoter enables the study of molecular circadian rhythms in vivo

## Introduction

The circadian clock controls biological processes such as behavior, gene expression, and physiology in diverse organisms, ensuring that these processes to take place at appropriate times of the day. This is crucial for many organisms, such as plants, which must synchronize photosynthesis with day-night cycles. Animals also synchronize to environmental cycles because of more subtle but nevertheless important needs such as predator avoidance, food availability, and optimal temperatures for various processes. Circadian clocks in all species share the following properties: They persist even in constant environmental conditions with periods near 24 h; they can be reset by environmental stimuli such as light and temperature; and their periods are relatively constant at different temperatures.

Recently, remarkable progress has been made in elucidation of molecular mechanisms of circadian clocks in diverse organisms. The common feature of clocks is cycling gene expression due to intracellular transcriptional feedback loops [1,2,3]. Genetic analysis in higher metazoan species, for example the fruit fly *Drosophila,* has been extremely valuable in identifying important players of the clockworks and their roles [4,5,6]. Identification of clock genes in *Drosophila* lead to molecular dissection of clock mechanisms in mammals mainly by testing whether homologs of *Drosophila* clock genes are involved in mammalian clocks. While this approach is informative, it harbors the risk of missing important factors that would have been found by forward genetic searches without preconceptions. Furthermore, gaps in our understanding of the clock mechanisms include factors responsible for the expression of positive transcription factors *Clock* and *Bmal* and mechanisms for clock protein turnover. In this regard, zebrafish is in a unique position as a vertebrate species in which large-scale forward genetic screens are convenient [7,8]. Furthermore, zebrafish circadian clocks have been shown to possess unique properties such as light entrainability of molecular rhythms in cultured organs and cells [9,10,11].

A behavioral screening for circadian mutations has been successfully carried out in zebrafish [12]. However, due to the limited capacity of this method, it is not suited to high-throughput screening. A method relying on bioluminescence rhythms mediated by luciferase reporting has been successfully used to screen for mutants affecting circadian gene expression in plants, cyanobacteria, and flies [13,14,15]. In vertebrates, however, luciferase reporting has been used mainly for recording circadian gene expression in cultured tissues and cells, because of technical difficulties in these species [16,17,18,19,20]. Bioluminescence rhythms mediated by a *zper4-luc* promoter fusion construct have been studied successfully in the zebrafish PAC-2 cell line [21]. While this approach was useful for promoter dissection, generation of transgenic animals is necessary for mutagenesis screening.

In this study, transgenic zebrafish were made in which cycling expression of the firefly *luc* gene is driven by the promoter of *per3* [22]. This promoter was chosen because *per3* mRNA has been shown to oscillate rhythmically in embryos as well as in a cell line [11,22]. For mutagenesis screening, it is most convenient and economical to test the youngest possible animals and avoid raising the animals that give negative results. In this regard, *per3-luc* was considered ideal for mutagenesis screening, because an in situ hybridization study showed that *per3* mRNA cycles starting on day 1 postfertilization with or without any entraining signals [22]. It was suggested that maternal *per3* mRNA present in the oocyte can set the phase of *per3* mRNA rhythms in early embryos. This result, however, is not consistent with other studies involving development of circadian rhythms: Rhythms of melatonin production require a light-dark (LD) transition later than 20 h postfertilization [23]; circadian swimming rhythms in larval fish develop during the first 4 d of development and require entraining signals late in embryonic development [24]; and rhythms of cell proliferation in larval fish develop only after exposure to several LD cycles [25].

In order to determine the earliest possible developmental time when rhythmic *luc* expression can be monitored in the *per3-luc* transgenic fish, embryos from the transgenic lines were monitored for bioluminescence from day 1 of development (Protocol S1). To our surprise, very low and non-oscillating levels of bioluminescence were detected during the first 4–5 d into development. Furthermore, consistent with the rhythms of melatonin production, locomotor activity, and cell division, rhythmicity of the *per3* gene expression gradually developed during the first several days postfertilization, and was observed only if fish were exposed to LD cycles during the hatching period or later. It was also found that ambient temperature affects *per3-luc*-mediated bioluminescence in a complex way. This study defines conditions under which the *per3-luc* transgenic fish can be used for mutagenesis screening and other types of studies.

## Results

### Generation of *per3-luc* Transgenic Fish

**Figure 1:**
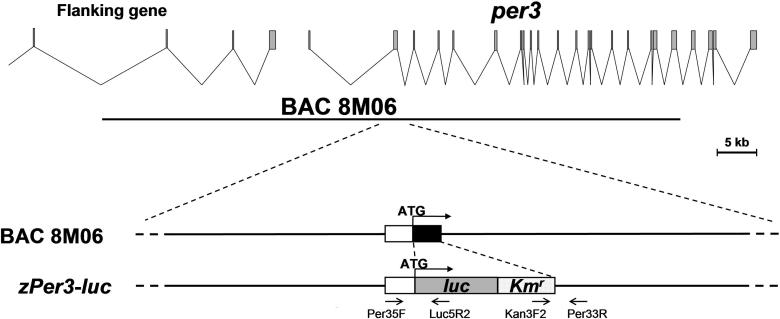
Schematic Map of the *per3-luc* Construct The top graphic shows the exon-intron structures of *per3* and the flanking gene. The BAC clone 8M06 screened for the first coding exon of *per3* is approximately 72 kb long, and extends from about 26 kb upstream of exon 1 to intron 19 of *per3*. The bottom graphic shows the magnified view of the first coding exon in the BAC 8M06 and the modified BAC construct. The white and black boxes represent noncoding and coding sequences, respectively, of the first coding exon of *per3*. The coding sequence of this exon was replaced with an approximately 3-kb fragment containing *luc* and *Km^r^*. Arrows under the construct represent primers used for the screening of transgenic lines.

To develop a system in which circadian gene expression in zebrafish can be monitored in vivo, transgenic fish were generated in which the expression of the firefly *luc* gene is driven by the promoter of the *per3* gene [22]. The construct was made by modifying a bacterial artificial chromosome (BAC) originally screened for sequences in the first coding exon of *per3* (Figure 1)*.* By comparison of *per3* cDNA sequence to the genomic sequence from another BAC clone (CH211–138E4) from this region, it was found that the cDNA contains another exon 5′ to the first coding exon. By comparing BAC-end sequences from the construct to genomic sequences from CH211–138E4 as well as with the Ensembl Zebrafish whole genome shotgun assembly sequence version 4 (http://www.ensembl.org/Danio_rerio/, the construct was found to be approximately 72 kb long, and spans from 26 kb upstream of exon 1 to intron 19 of *per3,* and contains part of another gene 5′ to *per3* (Figure 1). One canonical and two noncanonical E-boxes were found within 1 kb of the *per3* promoter (unpublished data). In the modified BAC, the coding portion of the first coding exon was replaced by the *luc* and *kanamycin resistance (Km^r^)* genes (Figure 1). This rather long construct was made because it has been shown in zebrafish that a reporter gene is more consistently expressed in a context of a longer BAC construct than in a conventional short construct with just a few kilobases of promoter sequences [26].

After screening 147 injected founders by PCR, five independent transgenic lines were found. Each positive founder was bred to a wild-type fish, and their progeny were individually tested for bioluminescence as 5–7-d-old larval fish. Larval fish with bioluminescence above background (more than 100 counts per second [cps]) were raised as transgenic F1 fish. Three of the five lines emitted bioluminescence above background. The level of bioluminescence varied depending on the line. The strongest-glowing line (#23) was used for this study unless otherwise stated. All the animals used in this study were the progeny of crosses between a transgenic line and the *AB wild-type strain. Therefore, these animals carried the transgene in hemizygous condition.

### Light Signals Are Necessary for *per3-luc* Rhythms

**Figure 2:**
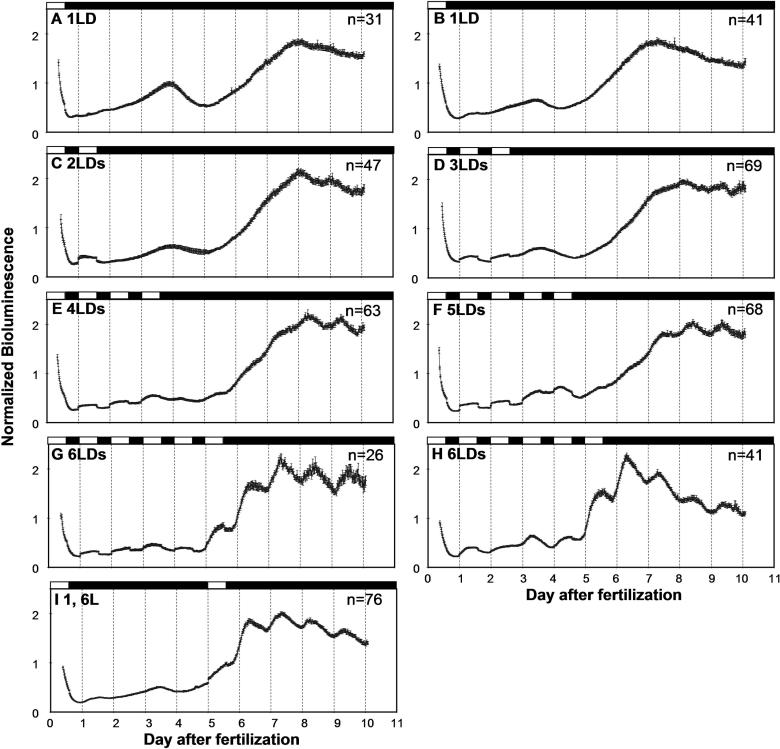
Bioluminescence in Embryos That Experienced Different Numbers of LD Cycles during Development Embryos hemizygous for *per3-luc* were collected and monitored for bioluminescence while exposed to different numbers of 14:10 LD cycles starting on day 1 postfertilization followed by DD. (A) One LD, (B) one LD, (C) two LD, (D) three LD, (E) four LD, (F) five LD, (G) six LD, and (H) six LD. In (I), embryos were exposed to two LDs, one on day 1 and the other on day 6 of development. Black and white bars on top of each plot represent the times when the lights were off and on, respectively. Since overall bioluminescence levels can vary among clutches and experiments, normalized bioluminescence was averaged and plotted in each graph. Number of animals that were averaged is given at top right corner of each plot. Error bars represent standard error of the mean (SEM) For the one-LD and six-LD groups, plots for two experiments are shown here. These experiments showed small differences in developmental profiles, possibly due to differences in room temperature (about 1 °C), therefore could not be pooled. For the two- to five-LD groups, data from two experiments were pooled. The small but abrupt increase of luminescence that occurred only during the light period of LD cycles is considered an artifact made visible by low bioluminescence counts during the first several days of development, because the same level of fluctuation was observed in empty wells under LD condition.

One of the intended usages of the *per3-luc* transgenic zebrafish is screening for mutations that affect bioluminescence rhythms. Since it is most convenient to screen the youngest possible animals, bioluminescence from transgenic embryos was monitored first. It was also expected that *per3-luc*-mediated bioluminescence in embryos should cycle from day 1 of development even in constant conditions, because *per3* mRNA expression detected by in situ hybridization has been demonstrated to oscillate from day 1 postfertilization in constant conditions [22]. Therefore, embryos carrying the transgene in hemizygous condition were collected and their bioluminescence monitored for 10 d starting from day 1 postfertilization. Surprisingly, when embryos were exposed to only one 14 h light: 10 h dark (14:10 LD, lights on at 8 A.M.; lights off at 10 P.M. CST) cycle on day 1, the majority of the animals showed no circadian rhythmicity of bioluminescence (Figure 2A and 2B; Table 1). Nevertheless, a characteristic developmental profile of *luc* expression was observed. Bioluminescence mediated by *per3-luc* stays rather low until day 4, when there is a small peak of bioluminescence, followed by a small dip on day 5 and a rapid increase that reaches the second peak on days 7–9. The slow decline of luminescence after that point may be due to substrate deprivation common in luciferase reporting [27]. Importantly, this developmental profile was also observed in two other lines of *per3-luc,* albeit with much lower overall luminescence counts (unpublished data).

**Table 1:**
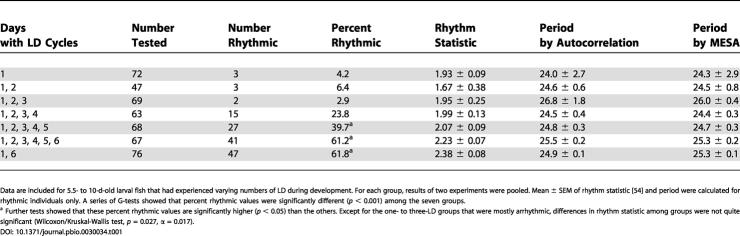
Rhythmicity and Periods of Larval Zebrafish: Varying Number of LD Cycles

Data are included for 5.5- to 10-d-old larval fish that had experienced varying numbers of LD during development. For each group, results of two experiments were pooled. Mean ± SEM of rhythm statistic [54] and period were calculated for rhythmic individuals only. A series of G-tests showed that percent rhythmic values were significantly different (*p* < 0.001) among the seven groups

^a^ Further tests showed that these percent rhythmic values are significantly higher (*p* < 0.05) than the others. Except for the one- to three-LD groups that were mostly arrhythmic, differences in rhythm statistic among groups were not quite significant (Wilcoxon/Kruskal-Wallis test, *p* = 0.027, α = 0.017)

Since the light signal on day 1 was not enough to elicit detectable rhythmicity of bioluminescence, an increasing number of LD cycles were given to embryos while they were monitored for bioluminescence (see Protocol S1). LD cycles on days 2 and 3 did not increase rhythmicity on subsequent days, although small fluctuations of bioluminescence were discernible on days 7–10 in the averaged plots (Figure 2C and 2D; Table 1). The number of animals expressing significant rhythmicity during the last 4.5 d of the record increased gradually when the number of LD cycles was increased from three to six (Figure 2D–2H; Table 1). The circadian fluctuation was superimposed on the developmental profile also seen in embryos entrained by fewer numbers of LD cycles (Figure 2).

To determine whether the number of LD cycles or the developmental stage at which the last LD transition occurred is more important for robust rhythmicity of luciferase reporting, embryos were monitored for bioluminescence while experiencing two LD cycles, one on day 1 and the other on day 6 (Protocol S1). Luminescence rhythms on the last 4.5 d of the record for this group of animals were as robust as those exposed to six LDs (Figure 2G–2I; Table 1). Thus, the developmental stage at which the last LD transition occurred, rather than the number of LD cycles, was important for light entrainment of *per3-luc* rhythms. There was no systematic effect of the time of the last lights-off on free-running periods (Tables 1 and 2).

**Table 2:**
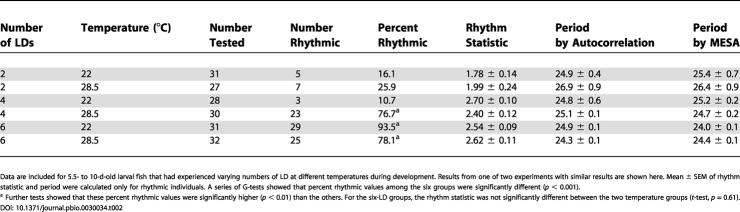
Rhythmicity and Periods of Larval Zebrafish: Varying LD and Temperature

Data are included for 5.5- to 10-d-old larval fish that had experienced varying numbers of LD at different temperatures during development. Results from one of two experiments with similar results are shown here. Mean ± SEM of rhythm statistic and period were calculated only for rhythmic individuals. A series of G-tests showed that percent rhythmic values among the six groups were significantly different (*p* < 0.001)

^a^ Further tests showed that these percent rhythmic values were significantly higher (*p* < 0.01) than the others. For the six-LD groups, the rhythm statistic was not significantly different between the two temperature groups (*t*-test, *p* = 0.61)

### Luciferase Reporting Reflects *per3* Expression

**Figure 3:**
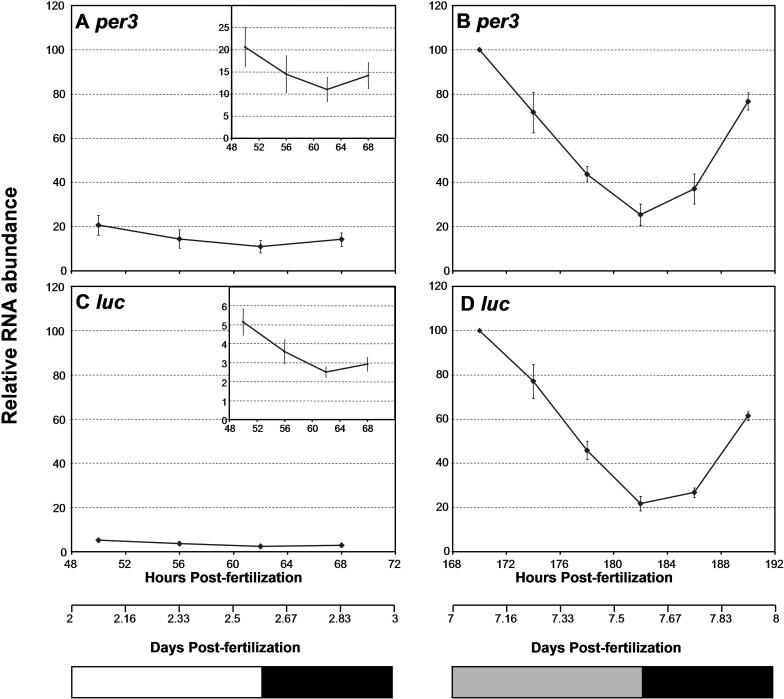
Temporal Expression of *luc* mRNA Is Similar to That of *per3* during Development Embryos hemizygous for *per3-luc* were collected from naturally breeding parents and kept in 14:10 LD cycles (lights on at 8 A.M. CST) at 22 °C for 6 d. Larval fish were shifted to DD at the end of the light phase on day 6. Total RNA was extracted from 2- to 3-d-old embryos and 7- to 8-d-old larval fish, and was subjected to real-time PCR for *per3* and *luc* mRNA levels. (A) Expression of *per3* mRNA per embryo on day 3 was determined every 6 h starting at 10 A.M. (2 h after lights-on). (B) The *per3* mRNA per animal on day 8 was measured every 4 h starting at 10 A.M. (C) Levels of *luc* mRNA on day 3 were determined as in (A). White and black bars at the bottom represent light and dark phases, respectively, for (A) and (C). (D) Cycling of *luc* mRNA on day 8 was determined as in (B). The gray and black bars represent the time when the light would have been on and off, respectively, had the LD cycles continued. For each of *per3* and *luc*, mRNA levels were normalized to the peak level on day 8 (10 A.M. time point). The y-axis scales were set at 120% maximum for all plots to allow direct comparison of mRNA extracted on days 3 and 8. The x-axis scales are given in both hours and days postfertilization to facilitate the comparison with Figure 2. In order to show more detailed temporal profiles of mRNAs on day 3, plots with smaller y-axis scales were shown in the insets at top right corners of (A) and (C). Each plot is the average of three identical experiments, and error bars represent SEM. An identical experiment was also done at 24 °C with essentially the same results (unpublished data).

The lack of bioluminescence rhythms during the first few days of development was rather unexpected because of the previously reported *per3* mRNA rhythms [22]. Therefore, mRNA cycling of *per3* and *luc* was compared by real-time quantitative PCR (qPCR). As in the experiment shown in Figure 2G and 2H, embryos were exposed to six LD cycles and transferred to constant darkness (DD). Embryos were collected and their mRNA was extracted on days 3 and 8. Both *per3* and *luc* mRNA levels were much lower on day 3 compared to day 8 (Figure 3). What appears to be approximately 2-fold oscillations of *per3* and *luc* mRNA on day 3 (see the insets on Figure 3A and 3C) were not statistically significant (*p* = 0.47 for *per3*, and *p* = 0.08 for *luc* by the Wilcoxon/Kruskal-Wallis test). In contrast, approximately 5-fold fluctuations of the transcripts were observed on day 8 (Figure 3B and 3D). This increase in overall expression levels and cycling amplitudes reflect the observed bioluminescence profile, although there were qualitative differences between bioluminescence and RNA as well as between *per3* and *luc* mRNA (see Discussion). Importantly, the peak phase of mRNA cycling on day 8 was 5–7 h advanced compared to the phase of bioluminescence cycling (compare Figure 2G and 2H to Figure 3B and 3D).

### Effects of Ambient Temperature on *per3-luc*-Mediated Bioluminescence

The experiments presented above were done at 21–24 °C simply because fish survived better at these rather low temperatures (see Materials and Methods). However, the previously documented circadian studies on zebrafish, including those involving development of rhythmicity, have been done mainly at the higher temperatures of 25–28.5 °C [22,23,24,25]. Since higher temperatures accelerate development in general, it was conceivable that development of bioluminescence rhythms may be faster at higher temperatures. However, it was not possible to do the same experiment at higher temperatures, because fish do not survive well in microtiter wells at temperatures higher than 25 °C. Therefore, embryos were raised in petri dishes at two different temperatures, 22 °C and 28.5 °C, while exposed to two, four, or six LD cycles. Subsequently, they were placed in microtiter wells and bioluminescence was recorded in DD at 21–24 °C (Protocol S1).

**Figure 4:**
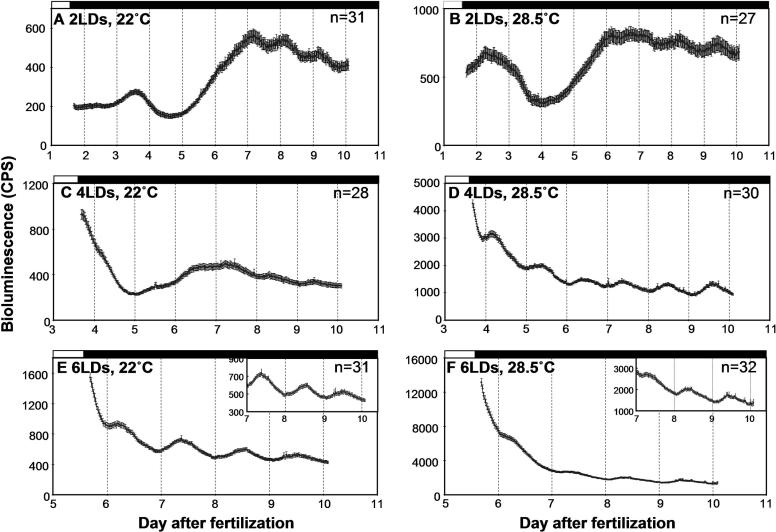
Effects of Temperature on Development of *per3-luc* Expression Transgenic embryos were entrained by two, four, or six LD cycles at either 22 °C or 28.5 °C, and monitored for bioluminescence in DD at 21–24 °C. (A) Two LDs at 22 °C, (B) two LDs at 28.5 °C, (C) four LDs at 22 °C, (D) four LDs at 28.5 °C, (E) six LDs at 22 °C, and (F) six LDs at 28.5 °C. The insets in (E) and (F) show the last 3 d of the record with magnified y-axis scales. Black and white bars on top of each plot represent the times when the lights were off and on, respectively. Actual amount of bioluminescence in cps is averaged and plotted in each graph. Number of animals that were averaged is given at top right corner of each plot. Error bars represent SEM. Results of one of two identical experiments with similar results are shown here.

The majority of embryos that were raised at 22 °C were arrhythmic after they were entrained by two or four LD cycles, but they were highly rhythmic after six LD cycles (Figure 4A, 4C, and 4E; Table 2). This is largely consistent with the trends observed in Figure 2 and Table 1, although more fish were rhythmic in this experiment for the two-LD and six-LD groups, and less in the four-LD group. The increased percentage of rhythmicity in this experiment for the six-LD group may be due to the fact that embryos raised in petri dishes are generally healthier than those raised in 96-well plates. Embryos raised at 28.5 °C showed significantly higher rhythmicity for the four-LD group than the same group raised at 22 °C (*p* < 0.05; Figure 4C and 4D; Table 2). This result shows that embryos raised at higher temperatures can be entrained earlier than those raised at lower temperatures.

In contrast to the effects on rhythmicity, temperature during the first several days of development seemed to have no systematic effects on periods: The two methods used for period estimation showed opposite effects of developmental temperature on periods (see the six-LD groups in Table 2). It should be pointed out that this may not mean that *per3-luc* rhythm is temperature compensated, because all the fish were monitored in the same temperature condition.

**Figure 5:**
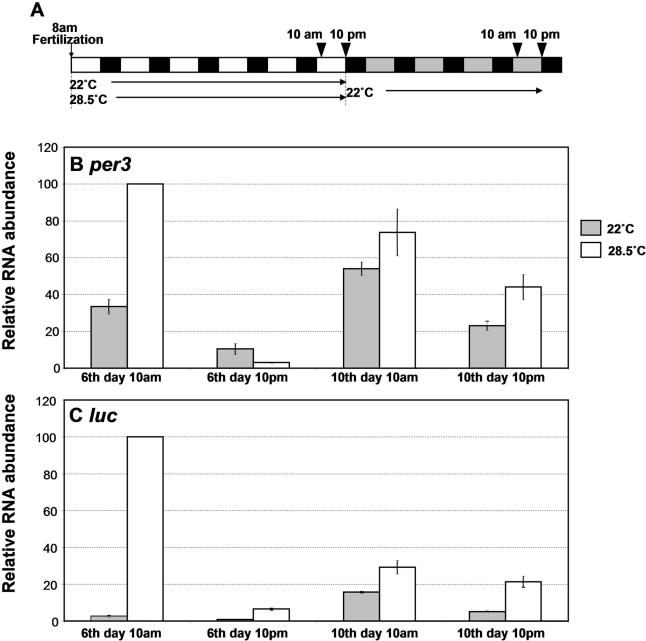
Levels of *per3* and *luc* mRNA Are Elevated by High Temperatures during Development (A) A schematic diagram showing how embryos were entrained and collected for RNA extraction. Embryos were entrained in 14:10 LD cycles (lights on at 8 A.M.; lights off at 10 P.M. CST) at two different temperatures, 22 °C and 28.5 °C. On day 6, half of the animals were sacrificed for RNA at 10 A.M. (2 h after lights-on) and at 10 P.M. (at lights-off). The rest of the animals were transferred to DD at 22 °C at 10 P.M. on that day, and sacrificed for RNA on day 10 at 10 A.M. and 10 P.M. The white and black bars represent day and night, respectively, and the gray bars the time at which lights would have been on had the LD cycles continued. The arrowheads indicate the time at which the animals were sacrificed for RNA extraction. (B) Relative mRNA level per animal for *per3* on days 6 and 10 of the experiment quantified by real-time qPCR. The levels were normalized to the value of the 10 A.M. time point on day 6 at 28.5 °C. (C) Relative RNA level per animal for *luc* measured from the same samples used in (B). For both (B) and (C), averages of three experiments are shown. Error bars represent SEM.

Besides development of rhythmicity, the developmental profile of *luc* expression and baseline level of bioluminescence were also affected by prior ambient temperature. For the two-LD groups, the first and second developmental peaks came earlier in the 28.5 °C than in the 22 °C group (Figure 4A and 4B). Higher temperatures also caused elevated levels of baseline bioluminescence, especially in the six-LD group (Figure 4E and 4F). This higher bioluminescence cannot be caused by high specific activity of equivalent luciferase enzyme, because all of the fish were monitored at the same temperature, and the difference in bioluminescence level persisted through over 4 d of monitoring. Therefore, this difference in luminescence most likely reflects a difference in the level of *luc* expression. Part of this difference between the two temperature groups may be explained by the fact that animals raised at higher temperatures are more mature and therefore express more luciferase than do those raised at lower temperatures. However, 10-d-old animals raised at 22 °C, which should have reached the plateau of luminescence (see Figure 2), showed much lower bioluminescence than 10-d-old fish raised at 28.5 °C (compare Figure 4E and 4F). Therefore, maturity of animals cannot explain the difference either. It seems that animals raised at higher temperatures simply express more luciferase than do those at lower temperatures. This was confirmed by a real-time PCR experiment (Figure 5). Cycling amplitudes and peak levels of *per3* and *luc* mRNA on day 6 were much higher in fish raised at 28.5 °C than those at 22 °C. The difference was especially large for *luc,* for which an approximate 40-fold difference between the two temperature groups was found at Zeitgeber Time 0 (2 h after lights-on; Figure 5C).

Bioluminescence in the high-temperature group gradually decreased over several days after they were transferred to lower temperatures, but did not fully return to the level of the low-temperature group (see Figure 4E and 4F). This is consistent with *per3* and *luc* mRNA levels determined by real-time PCR (Figure 5). Taken together, high temperatures elevate the level and cycling amplitudes of *per3* and *luc* mRNA, at least in larval fish, and this level and amplitude can gradually decrease after the animals are shifted down to lower temperatures.

### Optimal Condition for Recording Larval *per3-luc* Rhythms

**Figure 6:**
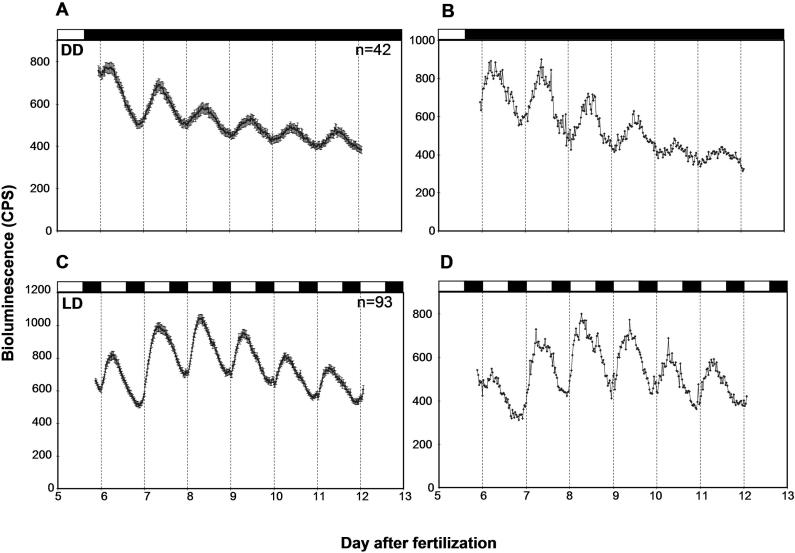
Bioluminescence Rhythms Mediated by *per3*-*luc* in DD and LD Measured for Six Days (A) Average plot of bioluminescence rhythms in DD. Animals were entrained in 14:10 LD cycles for 6 d at 22 °C and tested for approximately 6.5 d in DD. (B) Representative plot of bioluminescence rhythm in DD for an individual. (C) Average plot of bioluminescence rhythms in 14:10 LD cycles. Animals were entrained in 6 LD cycles at 22 °C prior to the monitoring. (D) Individual plot of bioluminescence rhythm in LD cycles. For each of DD and LD experiments, two experiments have been performed with essentially the same results. Only one of two experiments is shown for each of DD and LD. The first 12 h of data were deleted from each plot. Black and white bars on top of each plot represent the time when lights were off and on, respectively. Numbers of animals that were averaged is given at top right corner of (A) and (C). Error bars represent SEM in (A) and (C).

Fold amplitudes of bioluminescence rhythms were higher when embryos were entrained by LD cycles for 6 d at 22 °C rather than at 28.5 °C (see Figure 4E and 4F). Furthermore, survival of the animals was better if they were raised at 22 °C than at 28.5 °C (96.9% and 0% survival, respectively, on day 7). Therefore, embryos were entrained by six LDs at 22 °C in a petri dish, and tested for bioluminescence rhythms in DD (Protocol S1). In this experiment, embryos survived better than they did when they were placed in 96-well plates from day 1 onward (89.6% survival on day 12 in this experiment, compared to 71.5% on day 10 for the experiments presented in Figure 2). Furthermore, 88.1% (*n* = 42) of the fish were rhythmic with a 25.2 ± 0.7 h (mean ± standard deviation) period under this condition, and their rhythms persisted for 6 d, albeit with some damping (Figure 6A and 6B). In addition, *per3-luc* rhythms were tested in LD (Protocol S1). Amplitudes of bioluminescence rhythms in LD were higher than in DD, although they also damped slightly, possibly due to substrate deprivation (Figure 6C and 6D). A slightly higher percentage of fish was rhythmic in LD (95.0%, *n* = 179) compared to DD, although the difference was not significant (*p* > 0.1, G-test). The waveform of the rhythm in LD was different from that in DD: The ascending part of the wave that happens during the day was steeper in LD than in DD (Figure 6), suggesting that light may induce transcription of *per3*.

## Discussion

The *per3-luc* transgenic zebrafish system presented here is unique, because it is the only vertebrate system in which circadian gene expression in the whole animal can be studied in a high-throughput manner. This property of *per3-luc* in combination with zebrafish genetics makes the transgenic fish suitable for mutagenesis screening for circadian mutants. Embryos were tested initially, because it is more efficient to screen embryos than older animals, and movements of older animals can cause noise in bioluminescence signals [28]. However, the current study clearly demonstrates that monitoring embryos is not an option for any circadian studies using these lines. Of the conditions tested, raising fish to 6 d of age at 22 °C under LD cycles produced the most robust free-running rhythms. Rhythms measured under LD cycles were even stronger, and this condition has been used successfully in previous screens [15]. In addition to mutagenesis screening, it will facilitate other studies such as circadian organization of central and peripheral oscillators, entrainment pathways by various environmental cues, and physiological effects on circadian rhythms as have been studied in other organisms [17,18,29,30,31,32,33].

### Development of *per3* RNA Cycling in Larval Zebrafish

Larval fish that experienced an LD-DD transition later during development were more rhythmic than those shifted to DD earlier. This may mean that there is a critical developmental period after which *per3-luc* rhythms can be entrained. Alternatively, *per3-luc* rhythms might damp so fast that rhythms entrained a few days earlier could not be detected. We think the latter possibility unlikely for the following reasons: *per3-luc*-mediated bioluminescence rhythms persisted reasonably well for at least 6 d in DD if entrained properly (Figure 6A); at 28.5 °C, the rhythmicity of larval fish entrained for 4 d was comparable to that of fish entrained for 6 d, suggesting that bioluminescence rhythms do not damp in 2 d (Table 2); and *luc* mRNA cycling was almost undetectable during the first few days of development (see Figure 3C), and this low-amplitude fluctuation cannot give rise to high-amplitude oscillations unless there is a separate rhythm-amplifying mechanism operating during development, such as synchronization of cellular oscillators.

The results presented here, namely the gradual development of rhythmicity and responsiveness to entraining stimuli during the first several days of development and requirement of light signal, is consistent with previous observations on rhythms of melatonin production, locomotor activity, and the cell cycle in larval zebrafish [23,24,25]. It is also consistent with several studies in other vertebrate species involving gene expression [34,35,36] and physiological rhythms [37]. In both insects and mammals, free-running behavioral rhythms can develop normally in the absence of entraining signals, but their phases are not synchronized [38,39,40]. DeLaunay et al. [22] reported that *per3* RNA detected by in situ hybridization cycled synchronously from day 1 of development [22]. In our hands, however, *per3* and *per3*-driven *luc* RNA in 3-d-old larval fish detected by real-time PCR were not significantly rhythmic. Low-amplitude oscillations of both mRNAs may exist at this early developmental stage, because a similar trend (high in the morning) was observed in all three independent experiments done. However, this does not mean that the low-amplitude *per3* RNA oscillations that occur earlier during development can amplify into high-amplitude ones without any entrainment by LD cycles.

The increase of cycling amplitude over the course of several days of development in LD may happen within each cell that expresses *per3*. Alternatively, cycling amplitude may increase because various cellular oscillations present become synchronized. However, it was not only the cycling amplitude that increased with age, but the overall levels of *per3* mRNA also. Therefore, more cells may start expressing *per3* with high-amplitude oscillations later during development. This rather simple scenario is in fact what happens in developing *Drosophila;* as soon as the central pacemaker Lateral Neurons start expressing the PERIOD protein, the molecular rhythm is entrainable, and so is the eventual behavioral rhythmicity [40,41]. In any case, increasing amplitudes within cells, synchronization of oscillators, and more cells with high-amplitude oscillations are not mutually exclusive.

It is worth mentioning that rhythms of melatonin release starts as early as day 2 postfertilization, and this roughly corresponds to the time when the light-sensitive pineal gland is formed [23]. Another photosensitive organ, the retina, becomes photoresponsive as early as day 3 postfertilization [42]. Therefore, these organs become photosensitive, and/or develop rhythmicity prior to robust oscillations of *per3* RNA. Again, clocks in some tissues may develop earlier than in other tissues. It is also possible that *per3* may not be expressed in all the clock cells.

The biological significance of the developmental profile of *per3*-driven *luc* expression is not known. Most animals seemed to hatch between the first minor developmental peak and the subsequent trough (unpublished data). However, hatching itself is unlikely to induce *per3* expression in early embryos, because dechorionated *per3-luc* embryos showed developmental profiles of bioluminescence similar to those of nondechorionated siblings, albeit with an accelerated second rise of bioluminescence (unpublished data). Larval fish after the hatching period are supposed to have completed most of their morphogenesis and start swimming actively [43]. It may be that *per3* is important for rhythmic processes specific to hatched animals, such as behavioral rhythms [24].

### Consistency between *per3* Expression and Bioluminescence

The developmental and circadian profiles of *per3*-driven bioluminescence largely reflected endogenous *per3* expression. However, the amplitude of bioluminescence cycling was greatly reduced compared to that of *per3* or *luc* RNA. In general, proteins synthesized from cycling mRNAs show amplitude reduction and phase delay due to protein stability [44]. Consistently, luciferase-reporting studies in other organisms also showed dampening of cycling. For instance, in the *per*-*luc* transformants of *Drosophila,* approximately 6-fold cycling of *luc* RNA was reduced to 3- to 4-fold bioluminescence rhythms [27]. Similar reduction in cycling amplitude was observed for suprachiasmatic nuclei from the *per1-luc* mouse [18]. However, the reduction of amplitude was even more dramatic in *per3-luc* larval fish. This may mean that the luciferase protein is somehow more stable in larval fish than in flies or mice.

It should be noted that the luciferase protein itself is quite stable, but the enzymatic activity of this protein is unstable [28,45]. Therefore, the apparent stability of the luciferase protein in larval fish may be in fact stability of luciferase activity.

Besides the difference between bioluminescence and mRNA, there were differences between *per3* and *luc* mRNA, measured by real-time qPCR. The difference between days 3 and 8 of development was much larger for *luc* than *per3*. Furthermore, the difference between the 22 °C and 28.5 °C groups was larger for *luc* than for *per3*. These differences may be due to positional effect of the insert in the particular transgenic line used in this study. However, differences in bioluminescence levels between the first few days and older fish were found in two other independent *per3-luc* lines (unpublished data). Therefore, it is more likely that these differences between the two mRNA species reflect a property of the transgene itself. Although the BAC transgene used in this study has approximately 26 kb of upstream sequences, there may be critical sequences missing from this transgene, such as the first coding exon. Alternatively, posttranscriptional modification could be responsible for the difference. Posttranscriptional control of clock gene mRNA expression has been documented elsewhere [18,28,46,47].

### Effects of Ambient Temperature on *per3* Expression

Increasing ambient temperature had three effects on development of *per3* expression: It increased peak levels and cycling amplitudes of mRNA, and accelerated the developmental profile and development of responsiveness to the entraining stimuli. The latter two effects presumably are due simply to the fact that development is accelerated by higher temperatures. These results are not consistent with the report that development of a cell-cycle rhythm in larval fish was not affected by ambient temperature [25]. The increase in peak level and cycling amplitudes of *per3* mRNA induced by increased temperature is mostly independent of developmental speed. The peak *per3* mRNA level in the high-temperature group on day 6 was higher than that in the low-temperature group on day 10, when bioluminescence levels should have reached the plateau (see Figure 5). Ambient temperatures are known to affect levels of clock gene expression in *Drosophila* and *Neurospora* [47,48,49], and this could be the mechanism for clock resetting by temperature shifts [49]. Since our study was done on developing animals, it remains to be seen whether the increase in *per3* expression by elevated temperatures holds true in adults. Furthermore, it was not possible to test larval fish at 28.5 °C, and therefore it is not known whether the *per3* mRNA rhythm is temperature-compensated. Levels of *per3* and *luc* mRNA in fish raised at higher temperatures did not fully return to the levels in fish raised at lower temperatures, even after 4 d at lower temperatures. Therefore, there may be a mechanism for maintaining circadian periods despite change in *per3* expression levels.

## Materials and Methods

### 

#### Animals

Animals used in this study were derived from the University of Oregon *AB strain. Adults were kept under 14:10 LD (lights on at 8 A.M.; lights off at 10 P.M. CST) cycle, and group-housed in plastic tanks in a Z-MOD holding system (Marine Biotech) with recirculating filtered water at about 28.5 °C. They were fed commercial flake food in the morning, baby brine shrimp at midday, and adult brine shrimp in the evening. Experimental protocols were approved by the Institutional Animal Care and Use Committee.

Embryos were collected from naturally breeding fish in the morning, by plastic mesh traps that prevented parents from eating their progeny [23]. For microinjection, one- to two-cell-stage embryos were required. Therefore, male and female breeders were separated by a divider when they were placed in a trap. By removing the divider, fish were allowed to breed just before microinjection was performed [50].

#### Construction of *per3-luc* transgene

First, a zebrafish BAC library was screened for BACs containing 5′ coding sequences of *per3* [22] using a PCR-based screening kit (Incyte Genomics, Wilmington, Delaware, United States). One of two such clones, 8M06, was used for the construction of the transgene. The primers used for the screening were: forward, 5′- CCAGTAAAACGTCGTCGTCA-3′; reverse, 5′- GTCTGGGCCTGGAGAAGAGT-3′. The *per3* sequence from the initiation codon to the end of the first coding exon was replaced by a gene cassette containing the *luc* and *Km^r^* genes by homologous recombination in E. coli (see Figure 1) [51,52].

The *luc*/*Km^r^* gene cassette was constructed as follows. First, the *Km^r^* gene was cloned into NotI and SacI sites of pBluescriptSK+ (Stratagene, La Jolla, California, United States); then a HindIII-BamHI fragment containing *luc* and the SV40 polyA signal from pGL3-Basic (Promega, Madison, Wisconsin, United States) was cloned upstream of *Km^r^* into HindIII and BamHI sites of pBluescriptSK+; finally, a NotI site between *Km^r^* and the polyA signal was destroyed by digesting the clone with NotI and BamHI, followed by treatment with the Klenow fragment, and ligation of these blunted ends together.

Using this gene cassette as a template, a PCR fragment flanked by approximately 50-bp homology arms was amplified. The primer sequences used for the PCR reaction were: forward, 5′- GGGTTGTGAATCAGATCTTCAGTAGAGGAGGACAGGAGATCTCACAGGGAATGGAAGACGCCAAAAACATAAAGAAAG-3′; reverse, 5′- GTGCAGATTAAGTCAAATTCCACATAAAAAAAGCCACATTTCAAGTGTAC CGTTAATAATTCAGAAGAACTCGTC-3′. The forward primer contains 25 bp of sequence from the 5′ end of the *luc* coding sequences flanked by a 53-bp overhang corresponding to sequences just upstream of the initiation codon of *per3*. The reverse primer consists of a 50-bp overhang that corresponds to the intron sequences just downstream of the first coding exon, and 25 bp of sequence from the 3′ end of *Km^r^* as the primer.

The PCR fragment was purified and electroporated into DH10B cells containing the BAC and the plasmid pBAD-αβγ [51] as a source of recombinase genes. Cells in which the BAC was successfully modified were selected by kanamycin. The *luc* sequences in the modified BAC clones were checked for PCR errors by sequencing. One PCR error that resulted in a Val 217 to Ala change in the luciferase protein sequence was found. However, this is a conservative change, and fish injected with this construct showed bioluminescence above background. Therefore, it was judged that luciferase encoded by this construct can still function.

#### Generation of *per3-luc* transgenic lines

The *per3-luc* transgene was purified, linearized by NotI digestion, and injected into one- to two-cell-stage zebrafish embryos according to [53] with minor modifications. Injected embryos were raised to adulthood and individually bred to a wild-type fish or pairwise bred to each other. The progeny were tested for the presence of the transgene by PCR. PCR primers used for the screening were: Per35F, 5′- GCACCAGTAAAACGTCGTCA-3′; Per33R, 5′- TCATTCTCACTGGCAGAGCA-3′; Luc5R2, 5′- GTTTTAGAATCCATGATAATA-3′; Kan3F2, 5′- CTTTTTGTCAGAAGACCGACC-3′. The approximate positions of these primers with respect to the construct are shown in Figure 1. Transgenic fish were identified as those fish that gave an approximately 600-bp PCR product with the Per35F/Luc5R2 primer combination, and an approximately 800-bp product by the Kan3F2/Per33R combination, when their genomic DNA was used as templates. Nontransgenic fish gave no products with either of these primer combinations.

#### In vivo measurement of bioluminescence rhythms

Embryos or larval fish were placed individually in every other well of a white 96-well Optiplate (Perkin-Elmer, Wellesley, California, United States) with 200 μl of Holtfreter solution (7.0 g of NaCl, 0.4 g of sodium bicarbonate, 0.2 g of CaCl_2_, and 0.1 g of KCl [pH 7.0] in 2 l of ddH_2_O) aerated overnight and containing 0.5 mM D-luciferin potassium salt (Biosynth, Naperville, Illinois, United States) and 0.013% Amquel Instant Water Detoxifier (Kordon brand; Novalek, Hayward, California, United States). Once loaded with animals, four such plates were subjected to automatic monitoring of bioluminescence every 30 min by the Topcount multiplate scintillation counter (Perkin-Elmer) equipped with six detectors and plate stackers. The room temperature was set at 21–22 °C, and the machine at 24 °C. However, due to the heat created by the machine, temperature at the bottom of the stacker was 1–2 °C higher than the room temperature. In order to minimize high background counts under lighted conditions, each plate was dark-adapted for approximately 5 min before being counted for bioluminescence. Each well was counted for 4.8 s every 30 min. The plates were illuminated with two white fluorescent lamps, each facing the left or right side of the stacker. The approximate intensity of the light that reached the plates was 17–35 lux, depending on the position of the plates within the stacker.

#### Experimental protocol

The experimental protocol for each experiment involving in vivo bioluminescence measurement is described in Protocol S1.

#### Bioluminescence data analyses

Bioluminescence data from the Topcount were imported into Microsoft Excel 2000 by the Import and Analysis macro (kindly supplied by Steve Kay, Scripps Institute). In many of the experiments performed on the Topcount, some plates were placed in the machine several days earlier than others in order to monitor fish that experienced different numbers of LD cycles (Protocol S1). Therefore, at the end of the recording period that typically lasted for approximately 2 wk, many fish that were placed in the machine earlier had been dead for a few days. Since only the data up to 10-d old larval fish were analyzed for the experiments presented in Figures 2 and 4, simple observation of fish after the recording period could overestimate the number of fish that had died while the 10-d worth of data were collected. When a *per3-luc* fish dies in luciferin solution, it emits a burst of high bioluminescence counts (> 2,000 cps). This burst of luminescence is typically followed by a low background level of luminescence (< 50 cps). Furthermore, intermediate levels of spikes were also found in many plots just before the burst of high bioluminescence. Therefore, in order to eliminate data from dead fish, data that exceeded 5,000 cps, or those that went down below 50 cps, at any point of the analyzed portion of the data were first discarded. Then an averaged plot of the remaining data from each clutch of embryos was examined, and the highest count on days 8–9 was determined for experiments presented in Figure 2. For the other experiments, highest counts from the entire averaged plots except for day 1 of the record were determined. Then data that exceeded twice that value were also discarded as those with medium-sized spikes. This second round of data elimination was done in this way because overall luminescence counts varied among different clutches, possibly due to varying sizes of eggs laid. It should be noted that this procedure also eliminated records from fish that were alive, but that showed one or more transient spikes of bioluminescence. Those data with transient spikes were eliminated anyway, because the spikes can severely affect the accuracy of the data analysis program.

Period and rhythmicity for each animal were determined by a macro [54] based on MatLab 6.5 (Mathworks, Natick, Massachusetts, United States). With this macro, periods were determined by the maximum entropy spectral analysis (MESA) and autocorrelation, and rhythmicity by autocorrelation. Some fish that were apparently arrhythmic by visual examination of the plot gave rhythm indexes with a confidence interval higher than 95% by autocorrelation due to spurious peaks and small confidence intervals. Autocorrelation plots of these fish, however, were almost always nonsinusoidal and/or did not have five clear peaks (one at the center and two on each side of the center peak). Therefore, each set of data was judged blindly by three people as to whether its autocorrelation plot was sinusoidal with five peaks. Fish were judged as rhythmic only if two to three people found their autocorrelation plots sinusoidal, and their rhythm statistic values exceeded 1.

#### Real-time qPCR

Total RNA was extracted from 9–42 embryos or larval fish raised in petri dishes using TRIzol reagent (Invitrogen, Carlsbad, California, United States). The number of animals used for each extraction was recorded. Once extracted, total nucleic acid concentration was determined by a spectrophotometer. In order to prevent genomic DNA contamination, RNA samples were treated with Turbo DNA-free (Ambion, Austin, Texas, United States), and the concentration determined again by a spectrophotometer. Total RNA (0.5–1 μg) was subjected to cDNA synthesis by Superscript II Reverse Transcriptase (Invitrogen) using Oligo (dT)_12–18_ (Invitrogen) as the primer in a 25–40 μl reaction volume. Real-time PCR was performed in a 25 μl reaction volume containing a probe, forward and reverse primers, and qPCR Mastermix according to the manufacturer's instruction (Eurogentec, Seraing, Belgium). Each reaction was quadrupled in order to minimize pipetting errors. The primers and TaqMan MGB probes for *per3* and *luc* were designed and synthesized by the Assays-by-Design Gene Expression service (Applied Biosystems, Foster City, California, United States): *per3* forward, 5′- GCCCTGGCAGCACCA-3′; *per3* reverse 5′- GAAAGCTGGAGGACGAGGAA-3′; probe, 5′-6-FAM- CTAAGAGCTCAAAATCC-NFQ-3′; *luc* forward, 5′- GCAGGTGTCGCAGGTCTT-3′; *luc* reverse, 5′- GCGACGTAATCCACGATCTCTTTT-3′; probe, 5′-6-FAM- TCACCGGCGTCATCG-NFQ-3′. The ABI Sequence Detection System 7000 (Applied Biosystems) was programmed to perform the following protocol: 50 °C for 2 min, 95 °C for 10 min, followed by 40 cycles of 95 °C for 15 s and 60 °C for 1 min.

In this study, relative amount of *per3* or *luc* cDNA per animal was calculated by the standard-curve method [55] rather than by normalizing those RNA species to a constitutive control gene, for the following reasons: Both *per3* and *luc* were compared between two different developmental stages as well as among different times of the day. It was also important to calculate the amount of each mRNA species per animal in order to compare these data to bioluminescence data. The amount of a specific control RNA, as well as the total RNA, may differ among fish of different ages, in which case RNA per animal cannot be calculated by the relative quantification method using a constitutive control. As a concentration standard, a single-stranded DNA oligonucleotide of known concentration was used for each gene. These oligonucleotides span from the 5′ end of the forward primer to the 5′ end of the reverse primer, and including 75 bp for *per3* and 110 bp for *luc* (Biosource, Camarillo, California, United States). The standard concentration was varied from 10^2^ to 10^7^ copies per reaction in 10-fold increments. For every qPCR experiment, reactions for standards were performed in four replicates along with reactions for cDNA samples.

#### Statistics

To test whether percentages of rhythmic fish among different experimental groups were equal, the G-test was performed using Microsoft Excel 2000 according to Sokal and Rohlf [56]. If multiple tests were performed for a set of data, critical value of the G-statistic was adjusted for the experimentwise error rate [56]. For all the other numerical data, JMP 3.1.5 (SAS Institute, Cary, North Carolina, United States) was used for the following tests: Each set of data were first subjected to the test for normality. If the data were normally distributed, the one-way analysis of variance or the *t*-test was performed. The nonparametric Wilcoxon/Kruskal-Wallis test was performed on data that were not normally distributed even after various transformations (logarithmic, square root, and inverse) were tried. Where multiple tests were performed on a set of data, the experimentwise error rate (α) was adjusted by the Dunn-Sˇidák method [56].

## Supporting Information

Protocol S1 Experimental Protocol for Bioluminescence Experiments (27 KB DOC).

### Accession Numbers

The GenBank (http://www.ncbi.nlm.nih.gov/) accession numbers of the sequences discussed in this paper are *per 3* cDNA (NM_131584) and BAC clone CH211–138E4 (AL929204). The Ensembl (http://www.ensembl.org/Danio_rerio/) ID of the flanking gene of *per 3* mentioned in Figure 1 is ENSDARG00000023492.

## Abbreviations

BAC: bacterial artificial chromosome
cps: counts per second
DD: constant darkness
*Km^r^*: *kanamycin resistance* gene
LD: light-dark
*luc*: *luciferase* gene
MESA: maximum entropy spectral analysis
*per*: *period* gene
qPCR: quantitative PCR
SEM: standard error of the mean
