# Supplementary material for: Light-Dependent Development of Circadian Gene Expression in Transgenic Zebrafish
Source: PLoS Biol. 2005 Feb 1;3(2):e34. doi: 10.1371/journal.pbio.0030034 (PMC546037; doi:10.1371/journal.pbio.0030034)
Supplement: Protocol S1 — (27 KB DOC). [file pbio.0030034.sd001.doc]

**Protocol 1**

**Experimental protocols for bioluminescence experiments**

In the first set of experiments, bioluminescence of embryos was monitored from the first day to 10th day postfertilization during and after entrainment to different numbers of 14:10 LD cycles (1 to 6 LD cycles) starting on the day embryos were collected. In order to do this, embryos were loaded on a plate and monitored from the first day postfertilization with three other empty plates. Then another clutch of embryos was collected next day, or 2 to 5 days later in some cases, loaded on a plate, and monitored by replacing one of the empty plates. This was repeated until three to four plates were loaded with embryos. Then the light schedule was shifted to DD on the desired day at 10 pm. CST. In most experiments, embryos were not collected every day, because fish did not always breed, or in the case of later experiments, to monitor embryos that experienced desired numbers of LD cycles. All the plates were monitored until the last plate had been monitored for 10 days. Consequently, the first plate had been monitored for 13-15 days by the end of an experiment, and most fish in that plate were dead for a few days.

For the second set of experiments, embryos were monitored in the same way as above except for the lighting condition. Instead of consecutive LD cycles, they were given 1 LD on the first day and shifted to DD for 4 days. Then they were given another LD on the 6th day, and shifted to DD again until the end of the experiment on the 10th day.

In the third set of experiments, embryos were raised at two different ambient temperatures, 22˚C and 28.5˚C, while entrained by 2, 4, or 6 LD (14:10) cycles. Since monitoring at temperatures higher than 25˚C kills the fish, embryos were raised in petri dishes containing egg water while they were entrained, and subsequently monitored in the same condition as above in DD. In order to avoid variability among clutches, embryos from the same clutch were divided into two temperature groups, and entrained in LD cycles (35-40 lux). To test animals that experienced different numbers of LD cycles all at once, embryos were collected every other day. On the day after the embryos of the 2-LD groups were collected, embryos from all the groups were placed in the Topcount 1.5 h before the last lights off, and monitored in DD for 10 days.

The 4th set of experiments was done to test free-running bioluminescence rhythms in larval fish entrained for 6 days in 14:10 LD cycles. Embryos were collected one day and placed in petri dishes containing egg water, and entrained in LD cycles (35-40 lux) at 22˚C. Then they were monitored for bioluminescence for 7 days in DD.

The last experiment was performed to test bioluminescence rhythms in LD. Embryos were entrained as in the 4th experiment, and monitored in 14:10 LD cycles for 7 days. In order to subtract background luminescence counts, the last well for each column contained only luciferin solution but no fish.
